# Artificial Intelligence-based Radiomics in the Era of Immuno-oncology

**DOI:** 10.1093/oncolo/oyac036

**Published:** 2022-03-28

**Authors:** Cyra Y Kang, Samantha E Duarte, Hye Sung Kim, Eugene Kim, Jonghanne Park, Alice Daeun Lee, Yeseul Kim, Leeseul Kim, Sukjoo Cho, Yoojin Oh, Gahyun Gim, Inae Park, Dongyup Lee, Mohamed Abazeed, Yury S Velichko, Young Kwang Chae

**Affiliations:** Department of Internal Medicine, John H. Stroger, Jr. Hospital of Cook County, Chicago, IL, USA; Feinberg School of Medicine, Northwestern University, Chicago, IL, USA; Feinberg School of Medicine, Northwestern University, Chicago, IL, USA; Feinberg School of Medicine, Northwestern University, Chicago, IL, USA; Janssen Research and Development, LLC, Raritan, NJ, USA; Feinberg School of Medicine, Northwestern University, Chicago, IL, USA; Feinberg School of Medicine, Northwestern University, Chicago, IL, USA; Department of Internal Medicine, AMITA Health Saint Francis Hospital, Evanston, IL, USA; Department of Pediatrics, University of South Florida Morsani College of Medicine, Tampa, FL, USA; Feinberg School of Medicine, Northwestern University, Chicago, IL, USA; Department of Hematology and Oncology, Department of Medicine, University of Rochester Medical Center, Rochester, NY, USA; Feinberg School of Medicine, Northwestern University, Chicago, IL, USA; Department of Physical Medicine and Rehabilitation, Geisinger Health System, Danville, PA, USA; Department of Radiation Oncology, Northwestern University Feinberg School of Medicine, Chicago, IL, USA; Department of Radiology, Northwestern University Feinberg School of Medicine, Chicago, IL, USA; Department of Hematology and Oncology, Department of Medicine, Northwestern University Feinberg School of Medicine, Chicago, IL, USA; Robert H. Lurie Comprehensive Cancer Center of Northwestern University, Chicago, IL, USA; Department of Internal Medicine, Northwestern University Feinberg School of Medicine, Chicago, IL, USA

**Keywords:** radiomics, response prediction, machine learning, immunotherapy, tumor heterogeneity, immuno-oncology, automated intelligence, biomarker

## Abstract

The recent, rapid advances in immuno-oncology have revolutionized cancer treatment and spurred further research into tumor biology. Yet, cancer patients respond variably to immunotherapy despite mounting evidence to support its efficacy. Current methods for predicting immunotherapy response are unreliable, as these tests cannot fully account for tumor heterogeneity and microenvironment. An improved method for predicting response to immunotherapy is needed. Recent studies have proposed radiomics—the process of converting medical images into quantitative data (*features*) that can be processed using machine learning algorithms to identify complex patterns and trends—for predicting response to immunotherapy. Because patients undergo numerous imaging procedures throughout the course of the disease, there exists a wealth of radiological imaging data available for training radiomics models. And because radiomic features reflect cancer biology, such as tumor heterogeneity and microenvironment, these models have enormous potential to predict immunotherapy response more accurately than current methods. Models trained on preexisting biomarkers and/or clinical outcomes have demonstrated potential to improve patient stratification and treatment outcomes. In this review, we discuss current applications of radiomics in oncology, followed by a discussion on recent studies that use radiomics to predict immunotherapy response and toxicity.

## Teaser

Current methods for predicting immunotherapy response are unreliable and in need of improvement. This review discusses current applications of radiomics in oncology and recent studies that use radiomics to predict immunotherapy response and toxicity.

**Implications for Practice**

The current FDA-approved biomarkers for predicting immunotherapy response are programmed cell death-ligand-1 (PD-L1), tumor mutational burden (TMB), and microsatellite instability/defective mismatch repair (MSI/dMMR). Sampling through tissue biopsy, liquid biopsy, or cytology is required for immunohistochemical (IHC) detection of PD-L1 and dMMR, next-generation sequencing (NGS) analysis of TMB and MSI-high (MSI-H), and polymerase chain reaction (PCR) testing of MSI-H. However, the current sampling techniques often fall short of identifying individuals who will lack response, owing to inter- and intra-tumor heterogeneity in tumors. This review encompasses recent studies evaluating the application of radiomic models in cancer care, particularly in the era of immuno-oncology (IO). In summary, radiomic models may fill the current knowledge gap and allow for a more integrated and dynamic prediction of immunotherapy outcomes.

## Introduction

Advances in immuno-oncology (IO) are creating more options for patients with unresectable, metastatic, or chemorefractory malignancies lacking driver mutations. Decisions to treat patients with cancer using immunotherapy are often guided by molecular biomarkers that reflect a patient’s genetic background and immune profile. Unfortunately, up to 40% of patients do not respond consistently to immune checkpoint inhibitors (ICIs).^1^

The variable response to treatment has partially been explained by tumor heterogeneity, which is defined as the existence of distinct subclonal populations of cancer cells within a tumor.^2-4^ These subclones have different genetic profiles, resulting in differential protein expression that may render some cells resistant to immunotherapy.^5^ Biopsy has been used as a gold standard for predicting immunotherapy response. However, geographic heterogeneity is difficult to capture using biopsy because a single biopsy may not represent the entire tumor parenchyma.^6^ Additional challenges in cancer immunotherapy include cost, toxicity, and concerns over evaluating objective response. Various approaches based on clinical, molecular, immunologic, histologic, and radiomic profiles have been explored to account for tumor heterogeneity.

**Figure 1.**
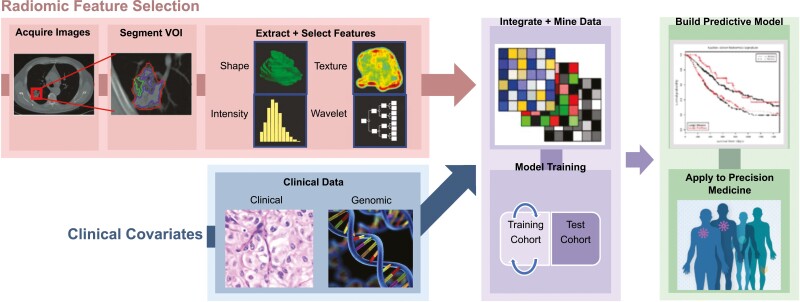
Radiomic model development. Radiomic models can be trained on many types of medical imaging. Images undergo segmentation to delineate ROI(s) and/or VOI(s) using a manual, semiautomatic or automatic approach. Feature extraction is performed on a training dataset using data processing software. Feature selection and model building are performed using machine learning methods to reduce redundant features, eliminate irrelevant features, and identify top features with high prognostic value. Clinical information can also be incorporated into model development. Model performance is measured using a validation dataset to assess overfitting and generalizability. Test datasets can be used to assess a final model fit. Performance is typically assessed in an independent validation dataset using AUC-ROC analysis, though studies lacking independent validation datasets may rely on internal validation techniques (random sampling, k-fold, bootstrap cross-validation, etc.).

Radiomics refer to the process of converting medical images into quantitative data (*features*), which can be mined to reveal complex patterns that reflect tumor biology at the macro- and microscopic levels. The process of constructing radiomics models is summarized in Fig. 1 along with Supplementary Appendix 1. The rationale behind this emerging technology is that disease processes at the molecular level manifest as distinctive macroscopic patterns on imaging that are difficult to discern by unaided visual assessment. Radiomic signatures (RS) built from chosen *features* can enhance clinical decision-making regarding cancer diagnosis, prognosis, and treatment. Artificial intelligence (AI) has improved radiomic model building by selecting the most salient radiomic features and incorporating clinical data into predictive models.

Radiomics has particularly important implications in cancer because of tumor heterogeneity. Compared to biopsy-based methods, radiomics can provide a more comprehensive assessment of the tumor by extracting features across the entire tumor microenvironment (TME). Additionally, radiomic models can be used on multiple scans, allowing clinicians to serially and non-invasively track changes in tumor phenotype and clinical response. In this review, we focus on applications of radiomics in IO, with special emphasis on studies that predict response to immunotherapy.

## Materials and Methods

Our review encompasses studies published before May 31, 2021, including full manuscripts, conference abstracts, presentations, and ongoing clinical trials. The search terms run in PubMed and EMBASE include “diagnosis,” “prognosis,” “treatment,” “chemotherapy,” “target therapy,” “therapy,” “immunotherapy,” and “immuno-oncology” in association with “radiomics.” We selected the studies based on their relation to oncology, use of AI, the inclusion of externally validated cohorts, validation metrics, cohort size greater than 100, and novel study designs as discussed in “future directions”. For the fairness of the assessment, we focus on studies that measure model performance by the area under the receiver operating characteristic curve (AUC) or the concordance index (CI).

## Applications of Radiomics in IO

Artificial intelligence-based radiomics has been investigated in many aspects of oncology (Supplementary Table S1, Tables 1 and 2). Radiomic models have demonstrated equally good performance as standard biopsy in correctly diagnosing and staging cancers.^7-24^ The models have also successfully predicted metastases, overall survivals, and regressions in various types of cancers.^25-41^ Most importantly, radiomic features have shown promise in treatment planning and evaluation.^42-98^

**Table 1.** Radiomics models trained on established biomarkers for cancer immunotherapy.

Reference	Tumor	Application	Train, validate, test (*n*)	Image	Performance^§^ (train, validate, test)
Sun et al.^66^	MST	Biomarker (CD8+ TIL^1^), Treatment (I; response, OS)	T 135 V1 (external) 119 V2 (internal) 100 V3 (internal) 137	CT	ML AUC T 0.74, V1 0.67, V2 0.76, V3 N/A
Li et al.^56^	Brain	Biomarker (TME^1^)	T 68 V (external) 56	MRI	DL AUC T 0.821, V 0.708
Tian et al.^68^	Lung	Biomarker (PD-L1^2^)	T 750 V (internal) 93 Ts (internal) 96	CT	ML AUC T 0.71, V 0.67, Ts 0.75 DL AUC T 0.63, V 0.67, Ts 0.68 Hybrid AUC T 0.78, V 0.71, Ts 0.76
Sun et al.^67^	Lung	Biomarker (PD-L1^2^)	T 260 V (internal) 130	CT	ML AUC T 0.786, V 0.807 ML-Combined AUC T 0.829, V 0.848
Jiang et al ^46^	Lung	Biomarker (PD-L1^2^)	T 266 Ts (internal) 133	PET/ CT	PD-L1 ≥1%: ML AUC T N/A, Ts 0.97 PD-L1 ≥50%: ML AUC T N/A, Ts 0.88
Yoon et al.^50^	Lung	Biomarker (PD-L1^2^)	T 153 V (internal) N/A	CT	ML CI T 0.550, V 0.550 ML-Combined CI T 0.667, V 0.667
Mu et al ^63^	Lung	Biomarker (PD-L1^2^), Treatment (I; DCB, PFS, OS)	PD-L1: T1 284 Ts1 (internal) 116 DCB: T2 99 Ts2 (internal) 47 PFS, OS: T3 146 Ts3 (prospective) 48	PET/ CT	PD-L1: ML AUC T1 0.89, Ts1 0.84 DCB: ML AUC T2 0.86, Ts2 0.83 PFS: ML CI T3 0.75, Ts3 0.79 OS: ML CI T3 0.79, Ts3 0.76
He et al.^45^	Lung	Biomarker (TMB^1^)	T 262 Ts (internal) 65	CT	ML AUC T 0.85, Ts 0.81 ML-Combined AUC T 0.75, Ts 0.74
Yoon et al ^73^	Lung	Biomarker (TIL^1^)	T 89 Ts (internal) 60	CT	Th1: ML AUC T 0.751, Ts 0.564 Th2: ML AUC T 0.795, Ts 0.684 CTL: ML AUC T 0.681, Ts 0.612
Yu et al. ^74^	Breast	Biomarker (TIL^1^)	T 85 V (internal)36	MG	TITreg: ML AUC T 0.83, V 0.79
Wen et al.^71^	Esophageal	Biomarker (PD-L1^2^, TIL^1^)	T 160 V (internal) 60	CT	PD-L1: ML AUC T 0.784, V 0.750 ML-Combined AUC T 0.871, V 0.692 CD8+ TIL: ML AUC T 0.764, V 0.728 ML-Combined AUC T 0.832, V 0.795
Gao et al.^54^	Gastric	Biomarker (TIL^1^)	T 90 V (internal) 45 Ts (internal) 30	CT	ML AUC T 0.884, V 0.869, Ts 0.847
Pernicka et al.^48^	Colon	Biomarker (MSI^2^)	T 139 Ts (internal) 59	CT	ML AUC T 0.74, Ts 0.76 ML-Combined AUC T 0.80, Ts 0.79
Liao et al.^57^	Liver	Biomarker (TIL^1^)	T 100 V (internal) 42	CT	ML AUC T 0.751, V 0.705
Chen et al.^43^	Liver	Biomarker (TIL^1^)	T 150 V (internal) 57	MRI^¶^	ML AUC T 0.904, V 0.899 ML-Combined AUC T 0.926, V 0.934
Iwatate et al.^55^	Pancreatic	Biomarker (PD-L1^2^)	T 107	CT	ML AUC T 0.683

All studies were retrospective unless otherwise specified.

Abbreviations: Tumor type MST: multiple solid tumors; Application CD: cluster of differentiation; DCB: durable clinical benefit; I: immunotherapy; MSI: microsatellite instability; OS: overall survival; PD-L1: programmed death-ligand 1; PFS: progression-free survival; TIL: tumor-infiltrating lymphocytes; TMB: tumor mutational burden; TME: tumor microenvironment; Train, validate, test T: training cohort; Ts: test cohort; V: validation cohort; Image CT: computed tomography; MG: mammography; MRI: magnetic resonance imaging; PET: positron emission tomography; Performance AUC: area under receiver operating characteristic curve; CI: concordance index, Combined: radiomics model combining handcrafted or DL features with clinical, radiologic, histologic, genetic, transcriptomic, proteomic, or metabolomic features; DL: deep learning-based radiomics; Hybrid: radiomics model combining handcrafted and DL features; ML: machine learning-based radiomics built on handcrafted features; N/A: not available.

Highest performing AUC and/or CI (other reported statistical analyses not included).

Peritumoral features included in analysis.

Assessed by next-generation sequencing

Assessed by immunohistochemistry

**Table 2.** Radiomics models trained on clinical outcomes of cancer immunotherapy.

Reference	Tumor	Application	Train, validate, test (*n*)	Image	Performance^§^ (train, validate, test)
Ligero et al.^58^	MST	Treatment (I; response)	T 115 V1 (internal) N/A V2 (external) 62	CT	ML AUC T 0.81, V1 0.72, V2 0.76 ML-Combined AUC T N/A, V1 N/A, V2 0.84
Dercle et al.^44^	Lung	Treatment (I, C, TT; response)	I: T1 72 V1 (internal) 20 C: T2 32 V2 (internal) 18 TT: T3 31 V3 (internal) 15	CT	I: ML AUC T1 0.80, V1 0.77 C: ML AUC T2 0.68, V2 0.67 TT: ML AUC T3 0.81 V3 0.82
Mu et al.^64^	Lung	Treatment (I; irSAE)	T 97 Ts1 (internal) 49 Ts2 (prospective) 48	PET/ CT	ML AUC T 0.88, Ts1 0.90, Ts2 0.86 ML-Combined AUC T 0.92, Ts1 0.92, Ts2 0.88
Vaidya et al.^70^	Lung	Treatment (I; HPD)	T 30 V (internal) 79	CT^¶^	ML AUC T 0.85, V 0.96
Tunali et al.^69^	Lung	Treatment (I; TTP, HPD)	T 228	CT	TTP <2 months: ML-Combined AUC T 0.812 TTP ≥2 months: ML-Combined AUC T 0.804 HPD: ML-Combined AUC T 0.843
Mu et al.^62^	Lung	Treatment (I; DCB, PFS, OS)	T 99 Ts1 (internal) 47 Ts2 (prospective) 48	PET/ CT	DCB: ML AUC T 0.86, Ts1 0.83, Ts2 0.81 ML-Combined AUC T 0.89, Ts1 0.86, Ts2 0.86 PFS: ML-Combined CI T 0.74, Ts1 0.74, Ts2 0.77 OS: ML-Combined CI T 0.83, Ts1 0.83, Ts2 0.80
Khorrami et al.^47^	Lung	Treatment (I; response)	T 50 V1 (internal) 62 V2 (external) 27	CT^¶^	ML AUC T 0.88, V1 0.85, V2 0.81
Liu et al.^59^	Lung	Treatment (I; response)	Baseline-radiomic dataset: T1 137 Ts1 (internal) 60 Delta-radiomic dataset: T2 112 Ts2 (internal) 49	CT	Baseline-radiomic dataset: ML AUC T1 0.59, Ts1 0.53 ML-Combined AUC T1 0.65, Ts1 0.61 Delta-radiomic dataset: ML AUC T2 0.82, Ts2 0.81 ML-Combined AUC T2 0.83, Ts2 0.81
Elkrief et al.^53^	Lung	Treatment (I; ORR)	T 141	CT	ML AUC T 0.67 DL-Combined AUC T 0.78
Yang et al.^72^	Lung	Treatment (I; response)	T 200	CT	60-day response: DL-Combined AUC T 0.77 90-day response: DL AUC T 0.69 DL-Combined AUC T 0.80
Mu et al.^61^	Lung	Treatment (I; cachexia, DCB, PFS, OS)	T 123 Ts1 (internal) 52 Ts2 (external) 35	PET/ CT	Cachexia: ML AUC T 0.77, Ts1 0.75, Ts2 0.74 ML-Combined AUC T 0.78, Ts1 0.76, Ts2 0.70 DCB: ML AUC T 0.71, Ts1 0.66, Ts2 0.70
Trebeschi et al.^49^	Lung, Skin (Melanoma)	Treatment (I; response)	T 133 Ts (internal) 70	CT	NSCLC: ML AUC T N/A, Ts 0.76 Melanoma: ML AUC T N/A, Ts 0.77
Lucas et al.^51^	Skin (Melanoma)	Treatment (I; PP)	T 112	PET/ CT	ML AUC T 0.78 ML-Combined AUC T 0.82

All studies were retrospective unless otherwise specified.

Abbreviations: Tumor type MST: multiple solid tumors; Application C: chemotherapy; DCB: durable clinical benefit; HPD: hyperprogressive disease; I: immunotherapy; irSAE: severe immune-related adverse event; ORR: overall response rate; OS: overall survival; PFS: progression-free survival; PP: pseudoprogression; TT: targeted therapy; TTP: time-to-progression; Train, validate, test T: training cohort; Ts: test cohort; V: validation cohort; Image CT: computed tomography; PET: positron emission tomography; Performance AUC: area under receiver operating characteristic curve; CI: concordance index; Combined: radiomics model combining handcrafted or DL features with clinical, radiologic, histologic, genetic, transcriptomic, proteomic, or metabolomic features; DL: deep learning-based radiomics; ML: machine learning-based radiomics built on handcrafted features; N/A: not available.

Highest performing AUC and/or CI (other reported statistical analyses not included).

Peritumoral features included in analysis.

Radiomics models carry insurmountable value especially in predicting systemic treatment response by way of noninvasive and comprehensive evaluation of the tumor. However, the aforementioned radiomics features require external validation ideally with datasets collected prospectively across  multiple centers.^44^

### Current Methods for Predicting and Evaluating Response to Immunotherapy

Treating patients with immunotherapy can be challenging due to a lack of reliable biomarkers for predicting and evaluating response.^99-102^ For example, an analysis of 45 PD-L1 FDA approvals from 2011 to April 2019 reveals that PD-L1 expression was predictive in only 28.9% of the approvals.^103^

The predictive ability of PD-L1, TMB, and MSI/dMMR remains uncertain largely due to the lack of standardized assays for determining biomarker status.^104^ Variability of commercial PD-L1 assays stems from the use of different PD-1/PD-1 antibodies, scoring systems, stained cells, cut-offs for PD-L1, and tumor types. Factors like the prevalence of MSI across tumor types as well as panel selection for IHC, PCR, and NGS contribute to the variability of MSI/dMMR testing. The large variation in TMB values is attributed to only limited attempts to standardize TMB calculation and reporting. Recent efforts by Friends of Cancer Research have yielded a TMB calibration tool that may resolve inter-assay variation; the tool has undergone 2 phases of analytical validation and will further require clinical validation to determine its clinical utility.^105^

Evaluating on-treatment response is also difficult given the current guidelines, namely Response Evaluation Criteria in Solid Tumors (RECIST), neither consider tumor structure nor appreciate immunotherapy-specific response events.^106^ Many patients receiving immunotherapy who experience atypical responses classified as progression by RECIST eventually show a durable and lasting response to therapy.^107,108^

Atypical responses to immunotherapy have been classified as pseudoprogression (PSPD), mixed response (MR), and hyperprogressive disease (HPD). PSPD demonstrates an initial enlargement of existing lesions and/or appearance of new lesions, followed by a delayed response and tumor shrinkage. MR characterizes a relative decrease in the size of existing lesions and the simultaneous appearance of new lesions. HPD is another poorly understood radiographic pattern observed in 9%-29% of patients treated with immunotherapy.^107^ This phenomenon reflects rapid disease progression after initiation of ICI therapy and often prompts treatment discontinuation.^107,109^

A number of modified criteria have been proposed to account for the novel patterns of immunotherapy response: immune-related response criteria (irRC), and immune-related RECIST (irRECIST), immune RECIST (iRECIST), and immune-modified RECIST (imRECIST)..^110-113^ PET-based assessments have also been proposed to account for metabolic and functional changes in tumors with greater sensitivity: PET Response Criteria in Solid Tumors version (PERCIST), the immunotherapy-modified PET Response Criteria in Solid Tumors (imPERCIST), PET Response Evaluation Criteria for Immunotherapy (PERCIMT), and Lymphoma Response to Immunomodulatory Therapy Criteria (LYRIC).^114-117^ Nonetheless, there exists no standardized evaluation tool that can be used consistently across clinical trials and in clinical practice.

Each of the preexisting criteria has some value. However, each possesses inherent limitations owing to the restricted metrics it employs. Artificial intelligence-based radiomics can potentially help to incorporate these varied metrics, including but not limited to tumor morphology and functional activity, thereby enhancing the predictive and prognostic power of the radiologic biomarkers.

### Radiomics for Predicting and Evaluating Response to Immunotherapy

Associations between RS and immunotherapy response can be determined using prediction algorithms built on existing biomarkers and/or clinical outcomes. Radiomic models can be trained to evaluate the established biomarker status, a measure that has traditionally been used to predict response to immunotherapy and guide treatment decisions. Thus, the RS is indirectly associated with response through biomarker status. In clinical outcome-based training, models learn to distinguish radiomic features that are directly associated with certain clinical outcomes.

Here, we highlight recent radiomic studies that assess immunotherapy response predictions according to the study approach (Table 2).

### Radiomics Models Trained on Established Molecular Biomarkers

Radiomics models can identify patients who will benefit the most from ICIs. The first machine learning (ML)-based RS capable of predicting PD-L1 expression is developed using CT, PET, or PET/CT scans from 399 patients with NSCLC.^46^ The model shows excellent performance in predicting over 1% and 50% expression in a test cohort (*n* = 133, AUC 0.97, 0.88, respectively).

While ML techniques generally require manual or semi-automatic extraction of features from *segmented* images, new approaches through deep learning (DL) can reduce the inconvenience. Deep learning enables direct use of *raw* images to automatically build a model and maximize its performance.^118^ Deep learning features can also train ML classifiers or merge with ML features to create a hybrid model. One study compares the models built using the ML or DL alone approaches with the hybrid of ML and DL approaches in their ability to identify patients with high PD-L1 expression.^68^ The radiomic features are extracted from pretreatment CT images of 939 NSCLC patients. The results demonstrate hybrid approach is superior to models built using the ML or DL alone. Its diagnostic efficacy is confirmed in training (*n* = 750, AUC 0.78, 0.71, 0.63 for hybrid, ML, and DL models, respectively), validation (*n* = 93, AUC 0.71, 0.67, 0.67), and test (*n* = 96, AUC 0.76, 0.75, 0.68) cohorts. Interestingly, the primary focus of the DL model lies in the peritumoral region and the textural differences between high and low PD-L1 expressions.

By integrating features from intra- and peritumoral regions, radiomic models may provide more reliable estimates of another important biomarker, tumor-infiltrating lymphocyte (TIL) abundance. Tumor-infiltrating lymphocytes indicate the robustness of immune response against a tumor and serve as strong prognostic indicators of clinical efficacy related to anti-PD-1 and anti-PD-L1 therapy.^119,120^ In a Chinese cohort study with 207 hepatocellular cancer patients, an MRI-based RS is trained to evaluate the density of CD3+ and CD8+ T-cells.^43^ The radiomic model, which includes features extracted from intra- and peri-tumoral regions, shows nearly excellent performance in a validation cohort (*n* = 57, AUC 0.899) and outperforms a model based on intra-tumoral features alone (AUC 0.639). Future studies may benefit from including peritumoral analysis, as this region is likely to hold prognostic value that enhances predictive ability.

A recent landmark study applies a radiomics model trained for TIL assessment in predicting clinical outcomes of immunotherapy.^66^ Using CT images and RNA-seq data evaluating *CD8B* gene expression from 135 patients with advanced solid malignant tumors, the study establishes and externally validates a radiomic biomarker of CD8+ T cells (*n* = 119, AUC 0.67, *P* =.0019). The study further validates its ability to discriminate between immune-desert and immune-inflamed phenotypes (*n* = 100, AUC 0.76, *P <* .0001). Higher radiomic scores are found to be associated with improved OS and objective response to anti-PD-1/anti-PD-L1 monotherapy (hazard ratio [HR] of 0.58, 95% CI 0.39-0.87; *P =* .0081). Although preliminary, this study serves as a foundation for subsequent research in radiomic analysis of the TME as a predictor of immunotherapy response.

Radiomic features can also capture various aspects of tissue heterogeneity that may be associated with the tumor genotype and genomic heterogeneity. As one example, a recent study identifies the TMB radiomic biomarker (TMBRB) using CT images from 327 patients with adenocarcinoma or squamous cell carcinoma.^45^ TMBRB shows good performance in distinguishing High-TMB (≥10 mut/Mb) and Low-TMB (<10 mut/Mb) in a test cohort (*n* = 65, AUC 0.81). Additionally, high- and low-risk groups established by TMBRB are shown to have significantly different OS and PFS (OS: HR 0.54, *P* = .03; PFS: HR 1.78, *P* = .023). Thus, a radiomics biomarker for TMB may provide insight into the genomic landscape of tumors which can guide treatment decisions.

Microsatellite instability (MSI) is another biomarker that may advance the current understanding of genetic variability and heterogeneous response to treatment. A radiomics biomarker for MSI status combined with clinical risk factors has shown efficacy in training and test cohorts (*n* = 139, 59; AUC 0.80, 0.79 for MSI).^48^ Preoperative radiomic identification of MSI colon cancers may shed light on patient stratification for neoadjuvant chemotherapy or immunotherapy (specificity 92.5%).

### Radiomics Models Trained on Clinical Outcome

The first radiomics-based models trained on clinical outcome utilize baseline and follow-up CT scans obtained after a 12-week course of anti-PD-1 therapy in 123 NSCLC and 80 melanoma patients.^49^ The RS performs well on individual NSCLC lesions (AUC 0.83, *P <* .001) but poorly on individual melanoma lesions (AUC 0.64, *P* = .05) likely due to prior exposure to other regimens and small cohort size. Performance varies widely among metastases at different anatomical locations in both cancer types, as imaging patterns may vary by anatomic site. When predictions from individual lesions are combined to assess patient-wide response, both cancers result in fair performance (AUC 0.76, *P* < .01). Certain texture and morphological features may be used universally to assess response; several features, including increased heterogeneity, non-uniform density, and compact borders, have been found to be associated with increased response regardless of organ or cancer type. Specificity of the RS for immunotherapy is confirmed in an independent validation cohort of 39 stage IV NSCLC patients treated with cytotoxic chemotherapy, as the RS fails to achieve significance in OS (*P =* .07) and response prediction (AUC 0.63, *P =* .09). Gene-set enrichment analysis performed externally in 262 NSCLC patients reveals radiomic association with cell division signaling pathways, suggesting that highly proliferative cancers may respond preferentially to immunotherapy.

Two recent studies identify an RS that can detect immunotherapy-related changes within the tumor earlier than volume-based assessment. One RS is built using peri- and intra-tumoral features extracted from CT scans acquired before and after 6-8 weeks of ICI therapy.^47^ In both internal and external validation cohorts, the model shows good performance in predicting response to therapy and OS (*n* = 62, 27; AUC up to 0.85). Perinodular features are significantly correlated with TIL density from tissue biopsy (*P* < .05). Furthermore, a model combining perinodular radiomic features and PD-L1 status (stratified by 50% criteria) better predicts OS compared to PD-L1 status alone. The radiomic model can differentiate between patient groups with different survival outcomes in earlier stages of treatment. In contrast, evaluating response based on tumor volume requires imaging acquired significantly later in treatment. Thus, integrating radiomic features reflective of immune activity with established immunotherapy biomarkers may improve models of response prediction and allow for earlier treatment evaluation.

Another study constructs a model, called iRADIOMICS, consisting of the radiomics features best predictive of immunotherapy response.^121^ Thirty patients with metastatic NSCLC treated with pembrolizumab are analyzed using baseline and follow-up PET/CT images. Radiomic multivariate analysis shows the highest performance using baseline images (AUC 0.90) as compared to baseline PD-L1 levels (AUC 0.60) and iRECIST at months 1 and 4 (AUC 0.79, 0.86, respectively). The standard iRECIST-based assessment requires to follow up images to monitor changes, resulting in a delay in clinical decision-making. In comparison, iRADIOMICS has the potential for pretreatment prediction of the ICI response from baseline imaging and therefore can lead to more efficient treatment planning.

Fused PET-CT may also serve as a novel basis for RS construction as it may offer complementary information in comparison to either imaging studies alone. A recent study with NSCLC patients extracts features from PET, CT, and Kullback-Leibler divergence images derived from fused PET and CT images.^62^ The resulting RS is predictive of durable clinical benefit (DCB) from ICI therapy in retrospective and prospective validation sets (*n* = 47, 48; AUC 0.83, 0.81, respectively). A combined clinical-radiomic model shows improved performance in both groups (AUC 0.86, 0.86, respectively). By capturing radiomic features reflective of tumor metabolism as well as tumor anatomy and shape, imaging multimodality allows a single radiomics model to generate a large volume of data linked to tumor heterogeneity. Therefore, enhanced assessment of heterogeneity can refine response predictions.

One multi-institution study takes a novel approach by linking cachexia with ICI resistance.^61^ The RS constructed from the features associated with cachexia in the PET/CTs of 210 ICI-treated NSCLC patients can identify those with DCB in training (*n* = 123, AUC 0.77, 0.71 for cachexia and DCB, respectively), test (*n* = 52, AUC 0.75, 0.66), and external test (*n* = 35, AUC 0.74, 0.70) cohorts. The high RS scores for cachexia are associated with shorter PFS and OS (*P < .*01), potentially due to cachexia-induced PD-1 downregulation. Furthermore, among the PD-L1 positive patients who are potentially sensitive to ICI, a low RS, defined as below median value of 0.04, correlates to longer PFS and OS (*P < .*01, *P = .*035, respectively). The results suggest that the cachexia RS can serve as a complementary prognostic marker to identify non-responders among PD-L1 positive patients. Further evaluation of the RS reveals that the features representative of heterogeneity increases the risk of cachexia and can also explain ICI resistance, as postulated previously.

Aside from the more typical tumor responses to therapy, which the current criteria can define more clearly, there exists a subset of atypical responses unique to immunotherapy. Pseudoprogression is rare and may even be underreported due to difficulty in differentiating it from true progressive disease (TPD). Therefore, PSPD still remains a retrospective diagnosis in the absence of validated response criteria.^107,112,122^ A pioneering study evaluates radiomics features from PET/CT to differentiate TPD from PSPD at an earlier time point at 3 month.^51^ The best performing model combines PSPD-associated features derived from imaging 112 metastatic melanoma patients with blood markers associated with OS in melanoma, LDH, and S100 (AUC 0.82). A subset of the analysis demonstrates that the features related to heterogeneous texture are more likely to represent true progression. These findings seem consistent with the current belief that tumor heterogeneity may breed resistance.

Hyperprogressive disease (HPD) is another atypical response that remains difficult to predict. A recent study designs a classifier indicative of HPD based on peritumoral radiomics features with a particular focus on the tumor blood vessels from pre-ICI treatment CTs of 109 advanced NSCLC patients.^70^ This DL algorithm predicts HPD from baseline imaging (AUCTest = 0.96) and displays prognostic significance by classifying HPD as having a worse OS compared to those without HPD (HR = 2.66, 95% CI 1.27-5.55; *P* = .0009). Interestingly, certain genetic mutations associated with HPD in previous studies, such as EGFR mutations and MDM2 amplification, were rare in this study and did not display any significant correlation with HPD. The results suggest further investigation into radiomic features in the TME may provide a novel approach to assessing the risk of HPD. Devising a robust AI-based model to predict atypical responses remains a challenge given the low incidence of PD and HPD. A large multicenter dataset is necessary to better define the role of radiomics as a predictive biomarker for atypical responses.

Training radiomic models on either biomarkers or clinical outcomes carries limitations in predicting response to immunotherapy. As numerous studies have already proven the predictive value of existing biomarkers, radiomic models based on molecular biomarkers can be expected to have some reliability. However, such models inherit biomarker-associated limitations and therefore fail to serve as a gold standard. Similarly, models that assess tumor response or progression as defined by preexisting guidelines fail to account for subvisual structural changes. These models can also suffer from a lack of model interpretability; without established biological or clinical logic behind the predictive power of such radiomic models, incorporating these preliminary models into the current treatment paradigm becomes challenging. Nonetheless, further validation and testing of such models can instill more confidence in radiomics models.

### Radiomics for Predicting Immunotherapy Toxicity

Immunotherapy-related adverse events (irAEs) can be severe and lead to premature treatment termination. A recent study reports that ≥2/3 of patients treated with anti-PD-1 or anti-PD-L1 experience a grade 3 or higher adverse event.^123^ Radiomics and other immunological biomarkers are being studied for the purposes of accessing the risk of irAEs.

One of the first algorithms predictive of checkpoint inhibitor pneumonitis (CIP) includes radiomics features representative of heterogeneous intensity.^124^ Although the training sample size is relatively small with only 2 NSCLC patients who develop CIP and 30 patients without CIP, the algorithm displays a strong predictive power (AUC 1.0, *P =* .0033).

Additionally, a novel preliminary RS is developed from pretreatment images of 9 NSCLC patients, who are later diagnosed with CIP.^125^ This radiomics model is tested on 42 cases without a clinical diagnosis of CIP and assigns 7 as greater than 50% probability of CIP; 6 out of the 7 misdiagnosed cases exhibit symptoms and radiologic signs of CIP based on chart review. Radiomics can help identify patients at risk of developing CIP with greater sensitivity than clinical findings alone.

Similarly, a radiomics nomogram incorporates immunotherapy type, dosage, and RS derived from pre-immunotherapy PET/CT images.^64^ The nomogram demonstrates the highest predictive value and overall net benefit across training, test, and prospective validation cohorts (*n* = 97, 49, 48; AUC 0.92, 0.92, 0.88, respectively) compared with RS (AUC 0.88, 0.90, 0.86) or clinical risk factors (AUC 0.74, 0.76, 0.68) alone. For clinical use, the aforementioned models will need to undergo refinement with multiple validations in varied cohorts. Nonetheless, the ability of radiomics to predict irAEs from pretreatment images alone shows a great promise in the field of IO as it may be tremendously cost-saving and even lifesaving in fatal cases.

## Limitations and Future Directions

Despite its immense potential, radiomics faces multiple challenges that ongoing studies attempt to tackle.

The lack of standardization in imaging studies remains a major limitation, as it may complicate data sharing and reduce the generalizability of models generated from institution-specific datasets. Additionally, nearly all radiomic studies have been retrospective analyses on small cohorts. Prospective, multicenter studies using larger cohorts are required for improved model validation and generalizability. As a proof of concept, one recent study collects and merges data from 3 multicenter datasets. The model built from the merged dataset performs better in classifying NSCLC phenotypes (AUC 0.78) than the models built from each of the 3 datasets separately.^21^ The expansion of medical imaging reservoirs combined with the development of increasingly advanced image analysis and pattern recognition technologies hold promise for improved model generation.

Overall, we believe the value of radiomics in predicting IO-related outcomes and adverse events can be summarized in its ability to generate integrated and dynamic models that provide insight into tumor biology.

### Integrated Model

**Figure 2.**
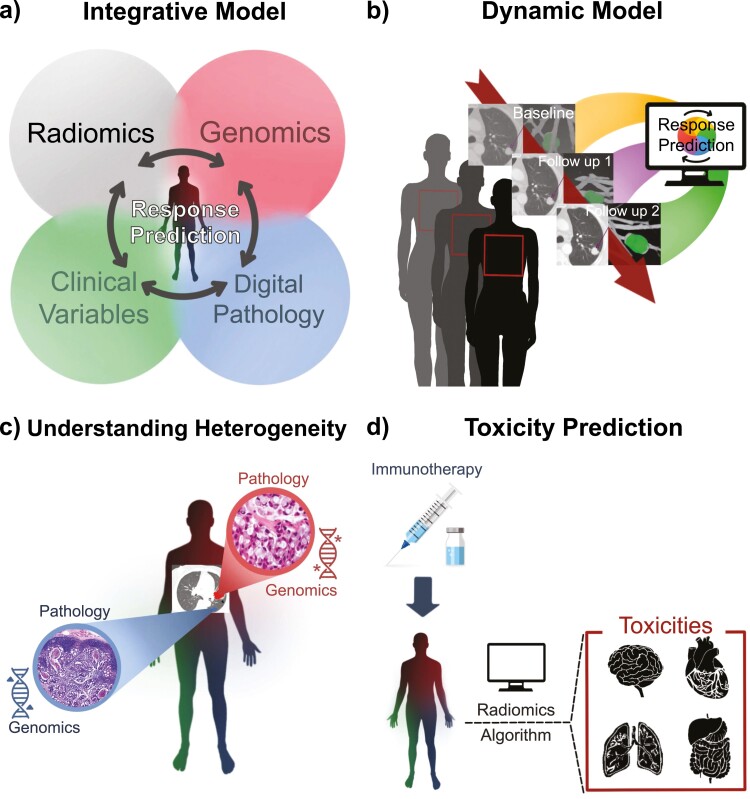
Radiomics as an Integrative and Dynamic Model and Future Applications. (A) Immunotherapy response predictions may benefit from models that integrate radiomic features with clinical, pathologic, and genomic information. (B) Radiomic models can non-invasively assess imaging studies performed during follow up visits, providing analyses that reflect spatial changes over time. (C) Radiomics can unravel the relationship between spatiotemporal heterogeneity of tumor burden and immunotherapy response, thereby allowing for improved immunotherapy strategies on an individual lesion and overall patient level. (D) Preliminary studies have shown radiomic models that identify patients at risk of developing an immunotherapy-related toxicity. Validation of these findings in future studies will allow clinicians to develop better treatment strategies.

Immunotherapy response is variable. Radiomic models are built using multiple features that reflect underlying biological processes, tumor heterogeneity, and pathophysiology. Future studies may investigate models integrating radiomic features with clinical, radiologic, histopathologic, genomic, transcriptomic, epigenomic, proteomic, and metabolomic information for optimal immunotherapy predictions (Fig. 2). As one example, the radiomics landscape is combined with a novel TME score that incorporates RNA sequencing or whole-exome data of 8210 immunotherapy-treated patients for OS prediction.^126^ The combined model (AUC 0.91) performs superior to unimodal predictors (AUC 0.75, 0.87 for radiomics landscape and TME score, respectively). The University Health Network (UHN) has also taken diverse approaches to define characteristics of the patients who develop primary or acquired resistance to immunotherapy (ClinicalTrials.gov. NCT04243720). The trial is in the process of collecting radiomic, genomic, transcriptomic, immunophenotypic, epigenetic, and fecal microbiome data, which can eventually be used to develop an integrated model to predict resistance.

### Dynamic Model

Immunotherapy response is dynamic. Evolutionary pressure from immunotherapy may promote the expansion of subclonal populations with increased treatment resistance. Radiomic models can non-invasively assess the TME at different timepoints. Particularly, delta-radiomics measures the spatial changes of radiomics features in response to treatment over time. The major limitation of delta-radiomics lies in the data collection. Because the frequency of screening during treatment differs by case, the validation of the delta-radiomics model becomes challenging. However, a recent study overcame the challenge through cross-validation and by utilizing a median predictive value for low and high-risk patients. Despite this limitation, radiomics still holds great promise, as features can be easily extracted from routine imaging studies performed during follow-up visits.

### Understanding Heterogeneity and Tumor Biology

Immunotherapy response is complex. Spatial and temporal fluctuations throughout tumors make it difficult to discern progression from atypical response patterns, for example.^6,127^ Understanding tumor heterogeneity will refine treatment strategy both on an individual lesion level and on a patient level. Numerous attempts have been made to indirectly measure heterogeneity through radiomics using texture analysis. However, applying radiomics alone will not suffice as feature reproducibility remains a major concern.^128^ Radiomic features vary widely across different tumor types, imaging modalities, and even institutions, each with its own contrast enhancement protocols that may affect the radiological texture. Selected features across existing studies do not usually overlap owing to inconsistent feature definitions, extraction, interpretations, and calculations. Incorporating biological features in RS building may strengthen the current knowledge of tumor heterogeneity but has not been done. An ongoing clinical trial strives to understand tumor heterogeneity through multi-omic analysis of the genomic, transcriptomic, epigenetic, immunophenotypic, and fecal microbiome profiles in patients with resistance to immunotherapy (ClinicalTrials.gov. NCT04243720).

### Toxicity Prediction

Immunotherapy response can be unpredictable. Toxicity related to ICI therapy is a major factor that precludes its further use. The search for improved predictive biomarkers of toxicity is ongoing. Notably, Maastricht University Medical Center in the Netherlands has been conducting a clinical trial to develop an AI-based radiomic model to identify the patients at risk of developing ICI-induced pneumonitis (ClinicalTrials.gov. NCT03305380). The prediction results can be factored into a cost-effectiveness analysis that will aid clinicians in making treatment strategies with greater precision.

Our institution is pursuing such an integrated approach to managing patients treated with immunotherapy. A protocol has been developed to identify a retrospective cohort of patients with available clinical data and to integrate the data with radiographic images, digital pathology, and genomic study from tissue and liquid biopsy. Multidisciplinary collaboration for data integration is underway. Future studies will focus on combining these factors (genomic, radiomic, and pathologic data) to build a unified predictive model that provides a more comprehensive analysis of tumor immune biology in response to immunotherapy (Fig. 2). Radiomics will serve as a powerful biomarker in this integrated prediction model that will promote the advancement of personalized medicine in IO.

## Supplementary Material

oyac036_suppl_Supplementary_Table_S1

oyac036_suppl_Supplementary_Supplement_1

## Data Availability

Data sharing is not applicable to this article as no datasets were generated or analyzed during the current study.
