# Supplementary material for: Artificial Intelligence-based Radiomics in the Era of Immuno-oncology
Source: Oncologist. 2022 Mar 28;27(6):e471–83. doi: 10.1093/oncolo/oyac036 (PMC9177100; doi:10.1093/oncolo/oyac036)
Supplement: oyac036_suppl_Supplementary_Table_S1 [file oyac036_suppl_supplementary_table_s1.docx]

**Supplementary Table S1.** Recent radiomics studies related to cancer diagnosis, prognosis, and treatment (chemotherapy, targeted therapy)

| **Reference** | **Tumor** | **Application** | **Train, Validate, Test (n)** | **Image** | **Performance**§ **(Train, Validate, Test)** |
| --- | --- | --- | --- | --- | --- |
| Zheng *et al.* [22] | Head and Neck  (Parotid gland) | Diagnosis | T 75  Ts (external) 52 | MRI | ML AUC T 0.926, Ts 0.902  ML-Combined AUC T 0.953, Ts 0.918 |
| Xu *et al.* [19] | Head and Neck  (Parotid gland) | Diagnosis | T 87  Ts (external) 38 | CT | ML AUC T 0.772, Ts 0.771  ML-Combined AUC T 0.854, Ts 0.835 |
| Liu *et al.* [15] | Head and Neck  (Parotid gland) | Diagnosis | T 74  V1 (internal) 35  V2 (external) N/A | MRI | ML-Combined AUC T 0.89, V1 0.85, V2 0.81 |
| Yang *et al.* [21] | Lungs | Diagnosis | Dataset 1:  T1 302  Ts1-1 (external) 203  Ts1-2 (external) 140  Dataset 2:  T2 203  Ts2-1 (external) 302  Ts2-2 (external) 140  Dataset 3:  T3 140  Ts3-1 (external) 302  Ts3-2 (external) 203  *Dataset 4:  T4 N/A  Ts4 (internal) N/A | CT | Dataset 1:  ML AUC T1 N/A, Ts1-1 0.58, Ts1-2 0.59  Dataset 2:  ML AUC T2 N/A, Ts2-1 0.57, Ts2-2 0.56  Dataset 3:  ML AUC T3 N/A, Ts3-1 0.58, Ts3-2 0.58  *Dataset 4:  ML AUC T4 N/A, Ts4 0.78  *Dataset 4 developed by merging Datasets 1-3, then randomly split into T4 and Ts4 |
| Shi *et al.* [18] | Lung | Diagnosis | T 219  V1 (internal) 135  V2 (external) 92 | CT | ML AUC T 0.805, V1 0.753, V2 0.792  ML-Combined AUC T 0.831, V1 0.792, V2 0.833 |
| Fan *et al.* [9] | Lung | Diagnosis | T 160  V1 (internal) 76  V2 (external) 75  V3 (external) 84 | CT | ML AUC T 0.917, V1 0.971, V2 0.942, V3 0.936 |
| Maldonado *et al.* [16] | Lung | Diagnosis | T 726  V (external) 170 | CT | ML AUC T 0.939, V 0.904 |
| Jiang *et al.* [13] | Breast | Diagnosis | T 278  V (external) 123 | US | ML AUC T 0.96, V 0.92 |
| Romeo *et al.* [17] | Breast | Diagnosis | T 135  Ts (external) 66 | US | ML AUC T 0.90, Ts 0.82 |
| Feng *et al.* [10] | Gastric | Diagnosis | T 90  V (external) 99 | CT | DL AUC T 0.988, V 0.929  DL-Combined AUC T 0.996A, V 0.932 |
| Cui *et al.* [8] | Pancreatic | Diagnosis | T 103  V1 (external) 48  V2 (external) 51 | MRI | ML AUC T 0.836, V1 0.811, V2 0.822  ML-Combined AUC T 0.903, V1 0.884, V2 0.876 |
| Wang *et al.* [24] | Renal | Diagnosis | T 122  V (external) 75 | CT | ML AUC T 0.89, V 0.81 |
| Zhou *et al.* [23] | Renal | Diagnosis | T 124  Ts1 (internal) 123  Ts2 (external) 73 | CT | Nuclear grade I:  ML AUC T N/A, Ts1 0.7141, Ts2 0.6936  ML-Combined AUC T N/A, Ts1 0.7743, Ts2 0.7487  Nuclear grade II:  ML AUC T N/A, Ts1 0.7315, Ts2 0.6610  ML-Combined AUC T N/A, Ts1 0.7535, Ts2 0.6767  Nuclear grade III:  ML AUC T N/A, Ts1 0.8132, Ts2 0.6562  ML-Combined AUC T N/A, Ts1 0.7857, Ts2 0.7264  Nuclear grade IV:  ML AUC T N/A, Ts1 0.8026, Ts2 N/A  ML-Combined AUC T N/A, Ts1 0.8492, Ts2 N/A |
| Castillo *et al.* [7] | Prostate | Diagnosis | Dataset 1:  T1 29  V1 (external) 78  Dataset 2:  T2 38  V2 (external) 69  Dataset 3:  T3 40  V3 (external) 67  *Dataset 4:  T4 86  V4 (internal) 21 | MRI | Dataset 1:  ML AUC T1 0.75, V1 0.55  Dataset 2:  ML AUC T2 0.69, V2 0.60  Dataset 3:  ML AUC T3 0.80, V3 0.65  *Dataset 4:  ML AUC T4 0.72, Ts4 0.75  *Dataset 4 developed by merging datasets 1-3 |
| Jian *et al.* [12] | Ovarian | Diagnosis | T 144  V1 (internal) 75  V2 (external) 75 | MRI | ML AUC T 0.899, V1 0.806, V2 0.847 |
| Li *et al.* [14] | Ovarian | Diagnosis | T 95  V1 (internal) 39  V2 (external) 26 | CT | ML AUC T 0.95, V1 0.96, V2 0.95 |
| Yan *et al.* [20] | Soft tissue | Diagnosis,  Prognosis (PFS) | T 109  V (external) 71 | MRI | Histopathological grade:  ML AUC T 0.846, V 0.829  ML-Combined AUC T 0.916, V 0.879  PFS:  ML CI T 0.568, V N/A  ML-Combined CI T 0.591, V N/A |
| Gitto *et al.* [11] | Soft tissue  (Cartilage) | Diagnosis | T 84  Ts (external) 36 | CT | ML AUC T 0.89, Ts 0.78 |
| Wang *et al.* [35] | Brain | Prognosis  (OS) | T 149  V (external) 66 | MRI | ML CI T 0.798, V 0.678  ML-Combined CI T 0.821, V 0.763 |
| Shen *et al.* [33] | Thymic | Prognosis  (OS) | T 96  V (external) 40 | CT | ML AUC T 0.78, V 0.74  ML-Combined AUC T 0.84, V 0.79 |
| Lee *et al.* [30] | Lung | Prognosis  (OS) | T 334  V (external) 47 | CT | ML-Combined CI T 0.753, V 0.778 |
| Yang *et al.* [39] | Lung | Prognosis  (OS) | T 239  V1 (internal) 80  V2 (external) 52 | CT | ML CI T 0.742, V1 0.726, V2 0.731  ML-Combined AUC T 0.747, V1 0.729, V2 0.710 |
| He *et al.* [28] | Lung | Prognosis  (M, RFS, OS) | T 268  V (external) 193 | CT | ML AUC T 0.75, V 0.73 |
| Ran *et al.* [32] | Lung | Prognosis  (M) | T 200  V1 (internal) 40  V2 (external) 60 | CT | ML CI T 0.775, V1 0.810, V2 0.844  DL CI T 0.777, V1 0.785, V2 0.812  Hybrid CI T 0.820, V1 0.830, V2 0.861 |
| Zhang *et al.* [41] | Breast | Prognosis  (M) | T 126  V1 (internal) 42  V2 (external) 62 | MRI | ML-Combined AUC T 0.82, V1 0.81, V2 0.81 |
| Wu *et al.* [37] | Esophageal | Prognosis  (M) | T 173  V1 (internal) 148  V2 (external) 90 | CT | ML-Combined CI T 0.725, V1 0.746, V2 0.728  CV-Combined CI T 0.798, V1 0.799, V2 0.791  DL-Combined CI T 0.875, V1 0.874, V2 0.840 |
| Xie *et al.* [38] | Esophageal | Prognosis  (DFS) | T 28  Ts1 (internal) 37  Ts2 (external) 41 | CT | ML AUC T 0.925, Ts1 0.782, Ts2 0.679  ML-Combined AUC T 0.912, Ts1 0.852, Ts2 0.769 |
| Zhang *et al.* [40] | Esophageal | Prognosis  (M) | T 130  V (external) 60 | PET | ML AUC T 0.69, V 0.63  ML-Combined AUC T 0.82, V 0.69 |
| Wang *et al.* [36] | Gastric | Prognosis  (R) | T 180  V (external) 144 | CT | ML AUC T. 0.88, V 0.90 |
| Shin *et al.* [34] | Gastric | Prognosis  (RFS, OS) | T 349  V (external) 61 | CT | RFS:  ML iAUC T 0.714, V 0.652  ML-Combined iAUC T 0.719, V 0.651  OS:  ML AUC T 0.733, V 0.878 |
| Bai *et al.* [26] | Renal | Prognosis  (M) | T 126  V1 (internal) 39  V2 (external) 36 | MRI | ML-Combined AUC T 0.914, V1 0.854, V2 0.816 |
| Bai *et al.* [25] | Prostate | Prognosis  (ECE) | T 158  V1 (internal) 68  V2 (external) 58 | MRI¶ | ML AUC T N/A, V1 0.678, V2 0.682  ML-Combined AUC T N/A, V1 0.718, V2 0.684 |
| Cuocolo *et al.* [27] | Prostate | Prognosis  (EPE) | T 104  Ts1 (external) 43  Ts2 (external) 46 | MRI | ML AUC T 0.83, Ts1 0.80, Ts2 0.73 |
| Hou *et al.* [29] | Prostate | Prognosis  (M) | T 180  V1 (internal) 71  V2 (external) 50 | MRI | ML AUC T 0.86, V1 0.83, V2 0.78  ML-Combined AUC T 0.92, V1 0.93, V2 0.72  DL AUC T 0.86, V1 0.84, V2 0.78  DL-Combined AUC 0.92, V1 0.91, V2 0.71  Hybrid AUC T 0.89, V1 0.85, V2 0.77  Hybrid-Combined AUC T 0.93, V1 0.92, V2 0.76 |
| Liu *et al.* [31] | Cervical | Prognosis  (M) | T 148  V1 (internal) 74  V2 (external) 51 | CT | ML AUC T 0.915, V1 0.782, V2 0.800  DL AUC T 0.912, V1 0.859, V2 0.800 |
| Park *et al.* [90] | Brain | Treatment  (S, C, RT; response) | T 238  V1 (internal) 93  V2 (external) 91 | MRI | ML AUC T N/A, V1 0.81, V2 0.65  DL AUC T N/A, V1 0.81, V2 0.78 |
| Zhai *et al.* [96]  Prior study [95] | Head and Neck | Treatment  (RT; nodal failure) | T 165  V1 (internal) 112  V2 (external) 113 | CT | ML CI T 0.84, V1 0.79, V2 0.71  ML-Combined CI T 0.90, V1 0.80, V2 0.71 |
| Song *et al.* [91] | Lung | Treatment  (TT; PFS) | T 145  V1 (external) 101  V2 (external) 96 | CT | 10-month:  DL AUC T 0.75, V1 0.72, V2 0.70  12-month:  DL AUC T 0.78, V1 0.73, V2 0.75 |
| Bousabarah *et al.* [75] | Lung | Treatment  (RT; LF, LC, DFS, OS) | T 110  Ts (external) 71 | CT | LF:  ML CI T 0.79, Ts 0.58  ML-Combined CI T 0.74, Ts 0.66  LC:  ML CI T 0.98, Ts 0.17  DFS:  ML CI T 0.97, Ts 0.52  OS:  ML CI T 0.99, Ts 0.45 |
| Cui *et al.* [80] | Lung | Treatment  (RT; RP, LC) | T 117  V1 (prospective) 25  V2 (external) 327 | PET | RP grade ≥2:  DL CI T 0.660, V1 0.667, V2 0.762  DL-Combined CI T 0.705, V1 0.691, V2 N/A  LC:  DL CI T 0.727, V1 0.706, V2 0.618  DL-Combined CI T 0.740, V1 0.721, V2 N/A |
| Jiang *et al.* [82] | Breast | Treatment  (C; pCR) | T 356  V (external) 236 | US | ML AUC T 0.89, V 0.92  Hybrid-Combined AUC T 0.95, V 0.94 |
| Liu *et al.* [60] | Breast | Treatment  (C; pCR) | T 128  V1 (external) 99  V2 (external) 107  V3 (external) 80 | MRI | ML AUC T 0.79, V1 0.70, V2 0.68, V3 0.79  ML-Combined AUC T 0.86, V1 0.79, V2 0.71, V3 0.80 |
| Hu *et al.* [81] | Esophageal | Treatment  (C; response) | T 161  Ts (external) 70 | CT | ML AUC T 0.901, Ts 0.805 |
| Niu *et al.* [88] | Liver | Treatment  (NS; P) | T 137  V (external) 81 | CT | ML CI T 0.723, V 0.734  ML-Combined CI 0.844, V 0.831 |
| Chen *et al.* [78] | Liver | Treatment  (NS; response) | T 355  V1 (internal) 118  V2 (external) 122 | CT¶ | ML AUC T 0.85, V1 0.81, V2 N/A  ML-Combined AUC T 0.96, V1 0.94, V2 0.90 |
| Li *et al.*  [84] | Prostate | Treatment  (S; bRFS, EPE, SVI, LNI, AP) | T 71  V (external) 127 | MRI | bRFS:  ML CI T N/A, V 0.77  EPE:  ML CI T N/A, V 0.70  SVI:  ML CI T N/A, V 0.82  LNI:  ML CI T N/A, V 0.77  AP:  ML CI T N/A, V 0.71 |
| Sun *et al.* [65] | Cervical | Treatment  (C; response) | T 183  Ts (external) 92 | MRI | ML AUC T 0.998, Ts 0.999 |
| Chen *et al.* [77] | Bone | Treatment  (C; pCR) | T 68  V (external) 34 | MRI | ML AUC T 0.882, V 0.842 |
| Wagner *et al.* [93] | Brain | Biomarker  (BRAF) | T 94  V (external) 21 | MRI | ML AUC T 0.75, V 0.85 |
| Choi *et al.*  [79] | Brain | Biomarker  (IDH) | T 727  Ts1 (internal) 129  Ts2 (external) 107  Ts3 (external) 203 | MRI | ML AUC T N/A, Ts1 0.90, Ts2 0.87, Ts3 0.84  DL AUC T N/A, Ts1 0.95, Ts2 0.91, Ts3 0.81  Hybrid AUC T N/A, Ts1 0.96, Ts2 0.94, Ts3 0.86 |
| Park *et al.* [89] | Brain | Biomarker  (IDH) | T 64  Ts (external) 57 | MRI | ML-Combined AUC T 1.000, Ts 0.863 |
| Casale *et al.* [76] | Brain | Biomarker  (1p/19q co-deletion) | T 159  V (external) 50 | MRI | ML AUC T 0.86, V 0.87 |
| Verduin *et al.* [92] | Brain | Biomarker  (IDH, MGMT, EGFR), Prognosis  (OS) | T1 72  T2 74  T3 64  T4 95  V (external) 38 | MRI | IDH:  ML AUC T1 N/A, V 0.695  MGMT:  ML AUC T2 N/A, V 0.667  EGFR:  ML AUC T3 N/A, V 0.707  OS:  ML-Combined CI T4 N/A, eV 0.711 |
| Zhang *et al.* [97] | Lung | Biomarker  (EGFR) | T 638  Ts1 (internal) 71  Ts2 (external) 205 | CT | ML AUC T N/A, Ts1 0.836, Ts2 0.778  ML-Combined AUC T N/A, Ts1 0.823, Ts2 N/A  DL AUC T N/A, Ts1 0.930, Ts2 0.642  Hybrid AUC T N/A, Ts1 0.910, Ts2 0.841 |
| Ninomiya *et al.* [87] | Lung | Biomarker  (EGFR) | T 99  Ts (external) 95 | CT | ML AUC T 0.86, Ts 0.77 |
| Mu *et al.* [86] | Lung | Biomarker  (EGFR) | T 429  V (internal) 187  Ts (external) 65 | PET/  CT | DL AUC T 0.86, V 0.83, Ts 0.81  DL Combined AUC T 0.88, V 0.88, Ts 0.84 |
| Li *et al.* [85] | Lung | Biomarker  (EGFR) | T 326  V (external) 112 | CT | Mutant/WT:  ML AUC T 0.77, V 0.76  ML-Combined AUC T 0.80, V 0.79  19/21 mutation site:  ML AUC T 0.74, V 0.71  ML-Combined AUC T 0.73, V 0.71 |
| Braman *et al.* [42] | Breast | Biomarker  (HER2),  Treatment  (TT; pCR) | T 42  V1 (external) 28  V2 (external) 50 | MRI¶ | Receptor status:  ML AUC T 0.80, V1 N/A, V2 N/A  Molecular subtyping:  ML AUC T 0.89, V1 N/A, V2 N/A  pCR:  ML AUC T N/A, V1 0.80, V2 0.69 |
| Zhang *et al.* [98] | Gastric | Biomarker  (Ki-67) | T 148  V1 (internal) 41  V2 (external) 150 | CT | ML AUC T 0.787, V1 0.765, V2 0.754  ML-Combined AUC T 0.801, V1 0.828, V2 0.784 |
| Jiang *et al.* [83] | Gastric | Biomarker  (TIL),  Treatment  (C; DFS, OS) | T 262  V1 (internal) 224  V2 (external) 106 | CT | ML AUC T 0.786, V1 0.745, V2 0.766 |
| Zeng *et al.* [94] | Renal | Biomarker  (VHL, BAP1, PBRM1, SETD2, molecular subtypes 1-4),  Prognosis  (OS) | T 104  V1 (internal) 103  V2 (external) 175 | CT | VHL:  ML AUC T N/A, V1 0.971, V2 N/A  BAP1:  ML AUC T N/A, V1 0.955, V2 N/A  PBRM1:  ML AUC T N/A, V1 0.972, V2 N/A  SETD2:  ML AUC T N/A, V1 0.949, V2 N/A  Molecular subtype 1:  ML AUC T N/A, V1 0.973, V2 N/A  Molecular subtype 2:  ML AUC T N/A, V1 0.968, V2 N/A  Molecular subtype 3:  ML AUC T N/A, V1 0.961, V2 N/A  Molecular subtype 4:  ML AUC T N/A, V1 0.953, V2 N/A  OS:  ML AUC T N/A, V1 0.775, V2 0.755  ML-Combined AUC T N/A, V1 0.846, V2 N/A |
| Cui *et al.* [52] | Rectal | Biomarker  (KRAS) | T 213  V1 (internal) 91  V2 (external) 86 | MRI | ML AUC T 0.722, V1 0.682, V2 0.714 |

All studies were retrospective unless otherwise specified. **Application/Prognosis** DFS: disease-free survival, ECE: extracapsular extension, EPE: extraprostatic extension, M: metastasis, OS: overall survival, RFS: recurrence-free survival. **Treatment** AP: adverse pathology, bRFS: biochemical recurrence-free survival, C: chemotherapy, LC: local tumor control, LF: local lung fibrosis, LNI: lymph node involvement, NS: non-surgical procedures, P: progression, pCR: pathological complete response, PFS: progression-free survival, RP: radiation pneumonitis, RT: radiotherapy, S: surgery, SVI: seminal vesicle invasion, TT: targeted therapy. **Biomarker** BAP1: BRCA1-associated protein 1 mutation, BRAF: B-Raf proto-oncogene status (fusions, V600E), EGFR: epidermal growth factor receptor mutation, HER2: human epidermal growth factor receptor amplification, IDH: isocitrate dehydrogenase mutation, Ki-67: nuclear protein Ki67 expression, KRAS: Kirsten rat sarcoma virus mutation, MGMT: O6-methylguanine DNA methyltransferase promoter methylation, PBRM1: polybromo-1 mutation, SETD2: SET domain containing 2 mutation, TIL: tumor-infiltrating lymphocytes, VHL: von Hippel-Lindau tumor suppressor mutation. **Train, validate, test** T: training cohort, Ts: test cohort, V: validation cohort. **Image** CT: computed tomography, MRI: magnetic resonance imaging, PET: positron emission tomography, US: ultrasound. **Performance** AUC: area under receiver operating characteristic curve, CI: concordance index, Combined: radiomics model combining handcrafted or computer vision or deep learning features with clinical, radiologic, histologic, genetic, transcriptomic, proteomic, or metabolomic features, CV: computer vision-based radiomics, DL: deep learning-based radiomics, Hybrid: radiomics model combining handcrafted and deep learning features, iAUC: integrated area under receiver operating characteristic curve, ML: machine learning-based radiomics built on handcrafted features.

§ Highest performing AUC and/or CI (other reported statistical analyses not included).

¶ Peritumoral features included in analysis.
