# Supplementary material for: Artificial Intelligence-based Radiomics in the Era of Immuno-oncology
Source: Oncologist. 2022 Mar 28;27(6):e471–83. doi: 10.1093/oncolo/oyac036 (PMC9177100; doi:10.1093/oncolo/oyac036)
Supplement: oyac036_suppl_Supplementary_Supplement_1 [file oyac036_suppl_supplementary_supplement_1.docx]

**Image Acquisition**

Radiomics can process many types of medical imaging (i.e. MRI, CT, PET). Medical images are essentially large data sets defined by thousands of 2D pixels or 3D voxels that can be mined by a radiomics classifier (*signature*) to identify features, which are patterns and relationships formed among data points that represent the image and reflect biological phenomena. Image quality depends on acquisition parameters (i.e. contrast administration, resolution, granularity, etc.) and 2D/3D reconstruction algorithms. However, imaging protocols differ among institutions and may lead to variation that more closely reflects the protocol rather than biology. [128, 129]. Recently, the Image Biomarker Standardization Initiative involving 25 research teams was able to standardize consensus-based reference values for 169 radiomic features [130]. However, challenges still remain in harmonizing the acquisition process.

**Segmentation**

Segmentation is the process of delineating regions or volumes of interest (ROI, VOI), which may include the entire lesion or subregions within or around the tumor. Segmentation is typically performed using computer-aided edge detection followed by manual curation by two or more experienced radiologists. Due to inter- and intraobserver variability, a semi-automated approach is most reliable and reproducible [131, 132]. Novel deep-learning segmentation technologies have been developed and are to outperform currently used semi-automated methods [133]. Segmented ROIs are then compiled and transformed into a 3D image, which reveals image features of increased complexity that may provide more insight into the underlying biological processes.

**Feature Extraction and Model Building**

Radiomic features are quantitative descriptors of imaging patterns that are classified as semantic or agnostic. Semantic features (size, shape, volume, etc.) refer to traditional radiographic descriptors that are commonly used in the radiology lexicon and can be identified by visual assessment. Agnostic features (entropy, haralick textures, wavelets, etc.) are quantitative descriptors that are derived using advanced statistical analysis on imaging data. Agnostic features can be defined by first-, second-, and higher-order statistical methods. First-order features describe overall distributions of data acquired from voxels in a given VOI without consideration for spatial relationships. These features are essentially single values that represent thousands of voxels in a VOI. Second-order features, or “texture” features, describe relationships between adjacent voxels as a function of contrast values. Higher-order features are obtained by applying mathematical transforms and filter grids to images and describe repetitive and non-repetitive patterns in voxel traits [128, 134].

Feature extraction is performed on a training (i.e. discovery, primary) dataset using data processing software and often provides thousands of features per image. Including a large number of features in a radiomic signature can lead to overfitting in which a model corresponds too closely with the training data set, leading to non-generalizability and poor performance using new data. To avoid the risk of overfitting, feature selection and model building can be accomplished through a variety of machine learning methods that reduce redundant features, eliminate irrelevant features, and identify top features with high prognostic value [129, 135].

References for Supplement 1

129. Gillies RJ, Kinahan PE, Hricak H. Radiomics: Images Are More than Pictures, They Are Data. Radiology. 2016;278(2):563-77.

130. Rizzo S, Botta F, Raimondi S, Origgi D, Fanciullo C, Morganti AG, et al. Radiomics: the facts and the challenges of image analysis. Eur Radiol Exp. 2018;2(1):36.

131. Zwanenburg A, Vallières M, Abdalah MA, Aerts H, Andrearczyk V, Apte A, et al. The Image Biomarker Standardization Initiative: Standardized Quantitative Radiomics for High-Throughput Image-based Phenotyping. Radiology. 2020;295(2):328-38.

132. Lee M, Woo B, Kuo MD, Jamshidi N, Kim JH. Quality of Radiomic Features in Glioblastoma Multiforme: Impact of Semi-Automated Tumor Segmentation Software. Korean J Radiol. 2017;18(3):498-509.

133. Owens CA, Peterson CB, Tang C, Koay EJ, Yu W, Mackin DS, et al. Lung tumor segmentation methods: Impact on the uncertainty of radiomics features for non-small cell lung cancer. PLoS One. 2018;13(10):e0205003.

134. Cardenas CE, Yang J, Anderson BM, Court LE, Brock KB. Advances in Auto-Segmentation. Semin Radiat Oncol. 2019;29(3):185-97.

135. Parekh V, Jacobs MA. Radiomics: a new application from established techniques. Expert Rev Precis Med Drug Dev. 2016;1(2):207-26.

136. Saeys Y, Inza I, Larrañaga P. A review of feature selection techniques in bioinformatics. Bioinformatics. 2007;23(19):2507-17.

137. Fave X, Zhang L, Yang J, Mackin D, Balter P, Gomez D, Followill D, Jones AK, Stingo F, Liao Z, Mohan R, Court L. Delta-radiomics features for the prediction of patient outcomes in non-small cell lung cancer. Scientific Reports. 2017;7(1):588.

138. Vega DM, Yee LM., McShane LM., Williams PM., Chen L., Vilimas T, et al. Aligning tumor mutational burden (TMB) quantification across diagnostic platforms: phase II of the Friends of Cancer Research TMB Harmonization Project. Annals of oncology. 2021;32(12):1626-36.
